# Supplementary material for: TLR4 competence and mouse models of sublethal leptospirosis
Source: PLoS Negl Trop Dis. 2025 May 30;19(5):e0013163. doi: 10.1371/journal.pntd.0013163 (PMC12151470; doi:10.1371/journal.pntd.0013163)
Supplement: Attachment — Submitted filename: Response to Reviewers MGS051525.docx [file pntd.0013163.s003.docx]

**RESPONSE to REVIEWERS**

**We would like to thank the reviewers for carefully reading and providing insightful comments to the manuscript. We used this opportunity to correct a few errors, clarify inconsistencies throughout and provide a manuscript that is substantially improved.**

**Major changes: we combined all data from 2 independent experiments, revised figures, did a new experiment (ELISA Procartaplex detection of 13 cytokines in serum from infected mice, Fig 5) and provide a substantial revision of the results and discussion.**

**Reviewer #1:**

**Summary and General Comments:**

Reviewer #1: This paper adds critical information to the field to try and separate the various mouse models of Leptospira infection that are in use. This is of extreme importance to furthering research and comparison between research groups that may use different models of infection. This is novel as no one has done a direct comparison of these mouse strains to date. Therefore, this is critical for the field. For convenience, I am copying all of my major and minor revisions below (which includes the experiments that need to be completed). Overall, I feel this paper should be published after the following revisions are addressed:

Methods Reviewer #1: The methods and objectives are clearly stated with a testable hypothesis. The study is appropriate. Concerns are listed below.

However, two issues are noted:
1. The authors are making the claim that C3H/HeJ mice are the only strain that sustained lethal infection, however, no comparison was ever made using age-matched mice of the other strains. Therefore this statement cannot be made without proper comparisons. Experiments need to be done with 7-8 week mice of the other strains to allow direct comparison.

We agree. We have evidence that mouse age at time of infection leads to full lethality (LD100) using an inoculum of 10^8^LiC in 7-8 weeks C3H-HeJ but we did not do a comparative analysis of lethal infection using age matched C3H-HeN. We resolved this issue by removing the LD100 infection in C3H-HeJ (7-8 week old) shown in Supplemental material. We clarified the age of mice used in Methods.

a. Line 31-32 – this experiment was done with the 7-8 week mice
b. Line 33-36 – This experiment was done with 9-10 week mice
c. The way lines 31-36 are written the authors state the C3H/HeJ mice had lethal infection, but then had similar survival curves - thus contradicting without stating these are different aged mice.
d. Apply this to other instances in the paper. Line 258-259, 276-277 etc.

Regarding survival, under LD50 (Fig 2), we included all data from 2 experiments showing that all mouse strains met endpoint criteria.

2. Mouse experiments were only done with 4 mice and two replicates. A third replicate needs to be done for accurate and valid comparisons to be made, as this is the standard for scientific conclusions to be drawn.

We agree that a total of 3 experiments is ideal. However, to comply with our institutional IACUC requirement of using the minimum number of animals to produce significant results, we use the Resource Equation approach to calculate nr of animals and we include a justification in Statistical analysis. Based on this approach, which is acceptable for exploratory animal research described in this manuscript, the acceptable range of degrees of freedom (DF) for the error term in an analysis of variance (ANOVA) is between 10 to 20 animals (Arifin, 2017). We have also used power calculation methods and arrive at the n=7-9 animals per group x 3 or 4 groups (acquired over 2 independent experiments) which we have been using in the lab for ~20 years. For this study, we clarify that in the first experiment we used 3 mice x 6 groups = 18 mice, and we repeated the experiment using 4 mice per group x 6 groups = 24 mice. In the revised version of this paper, we combined data from both experiments and describe it in results and new figs. We included this info in methods.

Results Reviewer #1: 1. Figures: Ideally, all three replicates (when the final one is performed) should be averaged and presented as a combined figure. Otherwise you need to provide a strong reason why you aren’t combining them, and include the data from all three in the supplement. This will allow reproducibility to be seen and verified.

**We combined all data from 2 experiments and** made new figures. We clarify in legends that “Data from two independent experiments is included” when applicable. We also include a Source file containing all data and statistical analysis.

2. Line 214 – an explanation of why younger mice were all of a sudden chosen for analysis is needed, and then as above, experiments need to be completed with the other strains with same ages of mice.

Lethal data with 7-8 week C3H-HeJ mice was generated before the start of this study and were removed from this paper.

3. Figures: Low quality images, when looking at it there seems to be pixelation causing additional faint lines to show up on graphs. Please provide better quality images.

Addressed.

4. Supplementary Figure 1: This would perhaps be best incorporated into Figure 1 so we can see the differences between the two ages of mice easily. This is an important conclusion you are drawing and it is in the supplement.

This figure was deleted.

5. Figure 3B: qPCR of a culture following a harvest does not provide quantitative information on the burden in tissue. Cultures do not grow the same and therefore this is not a quantitative measure and should not be included. You can include a statement that cultures were positive, which is what matters.

Lepto burden in tissue is shown in Fig 3A. For analysis of viability, we grow the Lepto out of the organ in EMJH medium and collect samples at 2 or 3 time points, d0, d4 and/or d7 for visual confirmation of motility under a dark field microscope and qPCR to provide quantitative information as a growth curve, which was not done in this case. In Fig 3B we show that we detect lepto on d4 of culture compared to the negative the control (which is zero) and we clarified the details of the method in M&M and in results.

Conclusions Reviewer #1: Generally, conclusions are supported. However, as described above there is no evidence that 7-8 week old mice of the C3H/HeN or C57BL/6 mice would not result in a lethal infection. This needs to be addressed experimentally in order for the conclusion that the C3H/HeJ is the only lethal model of infection (and the caveat added on age of mice).

We removed the 7-8 week old experiment from this paper and clarified the sublethal vs lethal issue throughout the manuscript.

In the discussion, a section needs to be added discussing the importance of the RT-qPCR results. These were only referenced in the results section with no discussion of their importance or differences between strains and what impact that would have on the model. Please add this to the manuscript.

A section (1pg) was included in discussion regarding RTqPCR and the new experiment on quantification of inflammatory markers in serum.

Reviewer #1: Minor (addressed with Yes, Y)
Line 26 – tlr4 is also expressed on other cell types, so I would remove “immune” from this line. Y
Line 30 – Remove “fully” Y
Line 80 – After [4] you need a “,” Y
Line 98 – Change “the LPS receptor” to “which recognizes LPS” as this receptor recognizes other ligands too. Y
Line 99 – Do you mean “One paper” and not “One mechanism”? Written this is confusing and I think an error. Y
Line 235 – Add statement about IL1-beta in C57/BL6 Y
Discussion – Reference figures when discussing data (such as line 266-267, 272, etc) Y
Line 274 – All mice were susceptible to “infection” equally as they were all infected. Change statement to reflect that C3H mice were more susceptible to “disease manifestations” or similar. Y
Figure 1 legend: “Weightloss” should be “weight loss” Y
Fix references to Figures in Text: There is no Figure 3C, yet it is ref in the text. Y

**Reviewer #2:** This manuscript presents a study comparing infection outcomes in TLR4-hyporesponsive (C3H-HeJ) and TLR4-competent (C3H-HeN, C57BL/6) mice. The study provides valuable insights into host-pathogen interactions, but several aspects require further attention before publication. The fact that C3H-HeJ mice serve as a good model for both sublethal and lethal leptospirosis has already been established in the field, diminishing the novelty of this study.

The major goal of the study was to evaluate if TLR4 competent C3H-HeN can be used as a mouse model of leptospirosis equivalent to TLR4 hyporesponsive C3H-HeJ, which continues to raise concerns of immunodeficiency as a mouse model due to TLR4 disfunction. We clarified this in the introduction.

We removed the data on C3H-HeJ 7-8 week lethal infections and will use it for a follow up comparative analysis between age-matched HeJ and HeN; this was a critical comment also made by the other reviewer.

Importantly, given the apparent low number of animals used, it is not possible to conclusively determine whether one mouse strain is more appropriate than another as a sublethal model of leptospirosis.

A major change done in this resubmission was to combine all data from 2 independent experiments and update the results section, stats, all the figures and discussion. To clarify, in the first experiment we used 3 mice x 6 groups = 18 mice, and we repeated the experiment using 4 mice per group x 6 groups = 24 mice.

To comply with our institutional IACUC requirement of using the minimum number of animals to produce significant results, we use the Resource Equation approach to calculate nr of animals and we include a justification in Statistical analysis. Based on this approach, which is acceptable for exploratory animal research reported in this paper, the acceptable range of degrees of freedom (DF) for the error term in an analysis of variance (ANOVA) is between 10 to 20 animals (Arifin, 2017). We have also used power calculation methods and arrive at the n=7-9 animals per group x 3 or 4 groups (acquired over 2 independent experiments, for reproducibility) which we have been using in the lab for ~20 years.

We don’t claim that one strain is better than the other. Rather, we claim that sublethal disease progression is not dependent on TLR4 competence. We highlight that in the title of the paper and specify that we present data for sublethal leptospirosis in mice.

**Methods:**

Reviewer #2: The manuscript clearly states its objectives; however, the hypothesis could be more explicitly defined.

We clarified the hypothesis in Introduction.

The design is generally appropriate for addressing the stated objectives, but the use of n=4 mice per group is a limitation, potentially affecting statistical power and reproducibility. The sentence in lines 120-121, which states "We used 4 animals per group, and experiments were reproduced once" is ambiguous. The authors should clarify whether they conducted two independent experimental replicates or only a single experiment without replication, which would be a major limitation.

See above regarding number of animals.

Additionally, the reasoning behind using both HAN and EMJH media should be explained.

We added the following in Bacterial strains… “We found that recovering -80C frozen Leptospira in HAN media before passage in EMJH allowed for faster recovery of the culture.”

The method of urine collection is not described and should be included in the methods section (lines 134-139).

“Immediately after the mice were restrained, the bladder was massaged, and Eppendorf tubes were used to collect the excreted urine.

The age of the mice used for cytokine analysis is unclear; lines 230-232 read as if a different experiment was conducted for this, which should be clarified in the methods.

The paper was substantially revised. Mice used in the experiments shown were all between 9-10 weeks old. We removed the 7-8 week old lethal experiment.

Furthermore, duplicates are usually not sufficient for qPCR analysis, and it is unclear how absolute quantitation of lipL32 copies was calculated.

Regarding the use of duplicates for qPCR analysis, we did two biological independent experiments to ensure the consistency of results and data reliability. We applied quality control measures, including proper normalization across independent experiments. Published studies in this field also use similar experimental designs with two biological replicates x 2 independent experiments. Regarding the absolute quantitation of Lepto DNA, we did not conduct lipL32 quantification but rather we amplify genome equivalents of 16S rRNA per mg of kidney tissue. Since we are looking for a bacterium in eukaryotic tissues, we don’t worry much about normalizing a bacterial gene against a eukaryote gene for analysis of pathogen burden (Fig 3) which we did for differences in genes from the same species (inflammatory markers in kidney after infection, Fig 4).

The term "confluency" in line 157 needs further clarification.

We replaced confluency with optimal cell density.

It is also unclear what tissue was used in the methods section for the expression of inflammatory and fibrosis markers.

We used kidney. This has been clarified.

The reasoning for choosing specific inflammation and fibrosis markers is not presented, and the method used for their quantification is questionable. A major advantage of mouse models over hamsters and guinea pigs is the availability of assays such as flow cytometry and ELISAs for direct evaluation of circulating cytokines in mice.

Analysis of circulating chemokines and cytokines and FACS immune phenotyping has been done for early infection (24h and 72h) in C3H-HeJ and that work has been published PMID: 34249775 and PMID: 35392072 cited. Repeating this in C3H-HeN would be subject of another study.

In this study we analyzed 9 inflammatory markers in kidney collected at euthanasia (15 days post infection). This has been clarified in results and Fig 4 legend. In the figure we present only the markers that show significant differences and added a comprehensive discussion on these results.

It is unclear why the study relied solely on RT-qPCR, a method also available for hamsters, despite its limitations.

To address this significant weakness, we did one more experiment that substantially improved this study. We measured 13 inflammatory marker proteins in serum from infected mice and show the new data in Fig 5 and added a section in discussion.

The statistical methods are appropriate but should be better justified in terms of their power to detect significant differences given the small sample size. The study adheres to ethical guidelines, but further clarification on humane endpoints and proper referencing of the literature would be beneficial to the reader.

We added a proper explanation of the statistical methods in the manuscript.

Regarding humane endpoints, we clarified in methods: “Mice were euthanized 15 days post-infection or when they reached the humane endpoint criteria (20% body weight loss).”

We revised the literature cited.

**Results:**

Reviewer #2: The results are presented in a logical order, but some aspects need clarification. The 75% survival rate mentioned in results (line 211) and in Figure 2 is unclear. If two experiments were performed, as suggested by the legend for Figure 2, but only one mouse died, then the percentage calculation is incorrect. To prevent this kind of misunderstanding, results for the presumed second experiment should be displayed.

To address this issue raised by both reviewers we now show the combined data from 2 independent experiments. This was especially important for accuracy of the survival curves in Fig 2.

In the paragraph in lines 203-213, the numbers in parentheses should be followed by an explanation of what they represent (leptospires?).

The paper was substantially revised.

Figures also need editing to improve data display. The simple timelines in Figures 2, 3, 4, and 5 are not necessary.

Timelines were removed and 300dpi TIFF files are now submitted.

The significance bars in Figure 3 should be adjusted to reduce the Y-axis, focusing on the data rather than dedicating excessive space to the significance bars. A similar issue is seen in Figures 4 and 5.

Weight loss data in Figure 1 could be combined, though this is a minor concern.

We combined data from 2 experiments and figures have been revised.

**Conclusions:**

Reviewer #2: The major limitations of this study are sample size and reproducibility.

See above regarding sample size and reproducibility. We appreciate these comments as they led to a substantial reanalysis of the data, and we are now much more confident about this revised manuscript.

The differences between infection outcomes in 9-10-week-old vs. 7-8-week-old C3H-HeJ mice (with only one 9-10-week-old animal dying while all 7-8-week-old animals died) are striking and underscore the need for a larger sample size.

We focus this paper on the comparative analysis of sublethal infection of C3H-HeN and C3H-HeJ at 9-10 weeks of age and removed the lethal analysis which was done previously in C3H-HeJ at 7-8 weeks old mice.

To provide meaningful conclusions, the study requires additional biological replicates (a total of at least two independent experiments) and infection experiments with decreasing doses of leptospires using all three mouse strains.

We did 2 independent experiments and are very confident about the data presented here for sublethal Lepto infection.

A study using age matched C3H-HeN and HeJ and decreasing doses of Leptospira would be interesting for our upcoming comparative analysis for lethal leptospirosis in C3H mice.

The discussion section could be improved, as many key references are missing. Review articles should be replaced with primary research articles where possible.

Agreed and addressed, cleaned up the literature.

**Editorial and Data Presentation Modifications?**

Reviewer #2 Minor The writing contains awkward sentence structures and grammatical issues that should be addressed for clarity.

The awkward sentences have been addressed.
